# Identification and validation of respiratory subphenotypes in patients with COVID-19 acute respiratory distress syndrome undergoing prone position

**DOI:** 10.1186/s13613-024-01414-y

**Published:** 2024-11-29

**Authors:** Mônica R. da Cruz, Pedro Azambuja, Kátia S. C. Torres, Fernanda Lima-Setta, André M. Japiassú, Denise M. Medeiros

**Affiliations:** 1 Evandro Chagas National Institute of Infectious Diseases, Oswaldo Cruz Foundation (INI-Fiocruz), Rio de Janeiro, RJ Brazil; 2 Pedro Ernesto University Hospital (HUPE), Rio de Janeiro State University (UERJ), Rio de Janeiro, RJ Brazil; 3 National Institute of Women, Children and Adolescents Health Fernandes Figueira, Oswaldo Cruz Foundation (IFF-Fiocruz), Rio de Janeiro, RJ Brazil

**Keywords:** Covid-19, Acute respiratory distress syndrome, ARDS, Subphenotypes, Mechanical ventilation, Machine learning

## Abstract

**Background:**

The heterogeneity of acute respiratory distress syndrome (ARDS) patients is a challenge for the development of effective treatments. This study aimed to identify and characterize novel respiratory subphenotypes of COVID-19 ARDS, with potential implications for targeted patient management.

**Methods:**

Consecutive ventilated patients with PCR-confirmed COVID-19 infection, in which prone positioning was clinically indicated for moderate or severe ARDS, were included in a prospective cohort. The patients were assigned to development or validation cohorts based on a temporal split. The PaO_2_/FiO_2_ ratio, respiratory compliance, and ventilatory ratio were assessed longitudinally throughout the first prone session. The subphenotypes were derived and validated using machine learning techniques. A K-means clustering implementation designed for joint trajectory analysis was utilized for the unsupervised classification of the development cohort. A random forest model was trained on the labeled development cohort and used to validate the subphenotypes in the validation cohort.

**Results:**

718 patients were included in a prospective cohort analysis. Of those, 504 were assigned to the development cohort and 214 to the validation cohort. Two distinct subphenotypes, labeled A and B, were identified. Subphenotype B had a lower PaO_2_/FiO_2_ response during the prone session, higher ventilatory ratio, and lower compliance than subphenotype A. Subphenotype B had a higher proportion of females (p < 0.001) and lung disease (p = 0.005), higher baseline SAPS III (p = 0.002) and SOFA (p < 0.001) scores, and lower body mass index (p = 0.05). Subphenotype B had also higher levels of the pro-inflammatory biomarker IL-6 (p = 0.017). Subphenotype B was independently associated with an increased risk of 60-day mortality (OR 1.89, 95% CI 1.51–2.36). Additionally, Subphenotype B was associated with a lower number of ventilator-free days on day 28 (p < 0.001) and a lower hospital length of stay (p < 0.001). The subphenotypes were reproducible in the validation cohort.

**Conclusion:**

Our study successfully identified and validated two distinct subphenotypes of COVID-19 ARDS based on key respiratory parameters. The findings suggest potential implications for better patient stratification, risk assessment, and treatment personalization. Future research is warranted to explore the utility of these novel subphenotypes for guiding targeted therapeutic strategies in COVID-19 ARDS.

**Supplementary Information:**

The online version contains supplementary material available at 10.1186/s13613-024-01414-y.

## Introduction

The COVID-19 pandemic has resulted in nearly 7 million deaths globally by July 2023 [1]. A severe complication of this disease is acute respiratory distress syndrome (ARDS), which is associated with high mortality rates [2]. A significant issue in the management of ARDS is the lack of effective treatments. The ARDS population is highly diverse, which is likely one reason why experimental therapies that showed promising results in preclinical studies have not succeeded in large clinical trials [3].

Prognostic and predictive enrichment have been proposed as strategies to improve the efficiency of clinical trials [4]. Individual respiratory parameters such as PaO_2_/FiO_2_, driving pressure, respiratory compliance, and ventilatory ratio provide valuable information about ARDS severity and prognosis [5–7]. However, relying on a single variable for phenotyping can be problematic due to the inherent heterogeneity of ARDS. For example, PaO_2_/FiO_2_, while useful, may not fully capture the complexity of respiratory mechanics and gas exchange abnormalities present in ARDS. This single-variable approach can lead to misclassification and an oversimplified understanding of the disease, ultimately limiting the effectiveness of targeted therapies [8].

To overcome these limitations, a more comprehensive view of the disease could be achieved by a multivariate subphenotyping approach considering the complex interplay between various disease characteristics. Such an approach could enhance our understanding of patient variability and help develop more personalized ARDS management strategies. Machine learning, a branch of artificial intelligence, uses algorithms to learn from data and make predictions [9]. It is valuable in medical research for identifying patterns that traditional methods might miss, which can be potentially helpful in understanding complex conditions like ARDS by analyzing multiple variables simultaneously [10].

The prone position reduces mortality in ARDS and has been widely used during the COVID-19 pandemic as a cost-effective procedure [11]. The prone position can improve oxygen exchange and promote a more uniform stress and strain distribution throughout the lung parenchyma by redistributing lung densities from the dorsal to the ventral lung regions. Furthermore, the prone position can increase lung recruitment, decrease atelectrauma, and improve ventilation-perfusion matching by reversing the gravitational forces that will enhance the expansion and ventilation of dorsocaudal regions [12]. Importantly, these effects can take place gradually over several hours during the prone session, and they are not homogenous among patients, reflecting the underlying patterns of lung injury [13]. Studies on COVID ARDS have indicated distinct prognostic implications of the oxygenation response to prone positioning and identified subgroups with unique respiratory profiles [14–16]. However, a significant heterogeneity in ventilatory mechanics poses a challenge to further understanding the specific features of COVID-19 ARDS [17]. Therefore, analyzing the joint trajectories of respiratory variables during the prone session can be a valuable strategy for the physiological classification of ARDS patients.

Interventions showing survival benefits in ARDS often rely on respiratory mechanics and the reduction of ventilator-induced lung injury (VILI) [18]. Previous studies on ARDS subphenotypes have focused on disease severity and inflammation, and only a few have focused on the crucial role of respiratory mechanics, especially in COVID-19 ARDS. Subphenotypes categorizing patients as recruitable and non-recruitable have been described in non-COVID-19 ARDS, relying on baseline respiratory variables, CT scan analyses, and gas exchange measures [19]. However, in COVID-19 ARDS, no difference in outcomes was found for those subphenotypes [20]. Additionally, an ascending trajectory of the ventilatory ratio (a marker of dead space ventilation) over several days was independently associated with a higher mortality in COVID-19 ARDS [21].

The aims of this study were, first, to identify and validate respiratory subphenotypes in patients with COVID-19 ARDS. For that purpose, we utilized the ratio of partial pressure of oxygen to the fraction of inspired oxygen (PaO_2_/FiO_2_), respiratory compliance, and ventilatory ratio as variables for clustering the patients. These variables were analyzed longitudinally before, during, and after the first prone session. Second, to explore the differences between the phenotypes regarding their clinical presentation, laboratory findings, and additional respiratory characteristics encompassing a broader set of variables. Third, to establish an association between the identified subphenotypes and patient outcomes.

## Methods

This study was approved by the local ethics committee and the Brazilian National Ethics Committee (CAAE:31050420.8.2001.5262), Brazilian clinical trials (RBR-2z3f7ke) as minimal-risk research using data collected for routine clinical practice and waived the requirement for informed consent. The procedures followed were under the amended Declaration of Helsinki of 1975.

### Study design and patients

We conducted a prospective observational study in a 120-bed Intensive Care Unit of COVID-19 Hospital Centre, a tertiary hospital built for the COVID-19 pandemic and part of Evandro Chagas National Institute of Infectious Diseases (INI), Oswaldo Cruz Foundation (Fiocruz), Rio de Janeiro, Brazil. We included all consecutive patients who met the following criteria: Age ≥ 18 years, a positive nasopharyngeal polymerase chain reaction (PCR) for SARS-CoV-2, a diagnosis of ARDS according to the Berlin definition [5], invasive mechanical ventilation, and a PaO_2_/FiO_2_ ≤ 200 mmHg, with a FiO_2_ ≥ 0.6 and a Positive end-expiratory pressure (PEEP) ≥ 5 cmH_2_O. The exclusion criteria were endotracheal intubation and mechanical ventilation for more than seven days on the day of the first prone session, any contraindications for the prone position, the need to interrupt the first prone session in less than four hours, and end-of-life decision. The patients were divided into development and validation cohorts by a temporal split, with admissions between 2020-05-27 and 2021-06-17 assigned to the development cohort and between 2021-06-18 and 2022-02-09 assigned to the validation cohort (Supplementary Fig. 1). The primary outcome was mortality at day 60. The secondary outcomes were ventilator-free days, being alive on day 28, and hospital length of stay.

### Data collection

Subject demographic and clinical baseline characteristics, including age, sex, body mass index, and comorbidities, were recorded. The Simplified Acute Physiology Score (SAPS) and the Sepsis-related Organ Failure Assessment (SOFA) were calculated to evaluate the severity of the disease. Arterial blood gases, ventilator settings, and respiratory variables: respiratory compliance, tidal volume, PEEP, respiratory frequency, minute ventilation, plateau pressure, driving pressure, and ventilatory ratio, calculated as (minute ventilation (ml/min) × PaCO_2_ (mmHg)]/(predicted body weight × 100 × 37.5). The respiratory and blood gas parameters were recorded at four time points: 1—“Baseline”, from the same day and before the start of the prone session; 2—“Early prone”, 1 h after the beginning of the prone session; 3- “Late prone” on the last hour of the prone session; 4—“Supine”, four hours after the termination of the prone session. Laboratory tests included C-reactive protein, D-dimer, procalcitonin, and IL-6 collected within 48 h of hospital admission. Each patient was followed until hospital discharge. Prior to analysis, the authors reviewed all data to identify and address any potential errors or incomplete entries. Study data were collected and managed using REDCap (Research Electronic Data Capture), an electronic data capture tool hosted at Evandro Chagas National Institute of Infectious Diseases.

### Statistical analysis

To identify the longitudinal subphenotypes, we used the PaO_2_/FiO_2_ ratio, respiratory compliance, and ventilatory ratio. The selection of these parameters was guided by their clinical and physiological relevance to ARDS pathophysiology, ensuring that they reflect meaningful differences in patient profiles. Furthermore, to maintain robustness and minimize redundancy, we avoided mathematically coupled parameters that might introduce bias or compromise the interpretability of the subphenotypes (Supplementary Fig. 2). Each parameter was available at four time points: Baseline, Early prone, Late prone, and Supine, as outlined above. Therefore, we included 12 data points per patient in the longitudinal clustering analysis: Respiratory compliance (Baseline), respiratory compliance (Early prone), respiratory compliance (Late prone), respiratory compliance (Supine), ventilatory ratio (Baseline), ventilatory ratio (Early prone), ventilatory ratio (Late prone), ventilatory ratio (Supine), PaO_2_/FiO_2_ ratio (Baseline), PaO_2_/FiO_2_ ratio (Early prone), PaO_2_/FiO_2_ ratio (Late prone), and PaO_2_/FiO_2_ ratio (Supine).

To obtain the subphenotypes from the development cohort, we utilized a k-means implementation designed specifically for joint trajectory analysis by handling multi-dimensional data as sets of variables (trajectories), using specialized distance metrics to capture their co-evolution, and standardizing trajectories to ensure equal contribution [22]. The distance metric was the Euclidean distance. The k-means algorithm ran 20 times with varying starting conditions, searching between 2 and 6 partitions. We used the Calinski–Harabasz criterion to determine the optimal number of clusters by comparing between-cluster and within-cluster dispersion. Higher values indicate better-defined clusters. We compared its values across different cluster numbers to find the best configuration, as this criterion is relevant only relative to other values. We also compared the longitudinal (4 time points feature set) with the features only at the Baseline timepoint using cluster stability analyses. We used the Calinski–Harabasz index to determine the number of clusters, and stability was assessed with 1000 bootstrap resampling runs [23]. The longitudinal feature set showed higher stability, perfect recovery, and no dissolutions, indicating a more robust clustering outcome (Supplementary Tables 1 and 2). This confirmed our preference for the longitudinal set. Sensitivity analyses were performed using only the complete observations and only participants with a baseline PaO_2_/FiO_2_ < 150 from the development cohort.

To investigate the reproducibility of the subphenotypes, we trained a random forest model using the k-means-labeled dataset [24, 25]. The number of trees was 500, with three variables tried at each split. The out-of-bag error rate was used to assess the model’s performance on unseen data unbiasedly. The importance of each variable for the classification was estimated by the Mean Decrease Accuracy and the Mean Decrease Gini. The random forest model was then used to classify the validation dataset. The demographic, clinical, and outcome data were obtained for comparison with the development cohort.

The survival rates were accessed using the Kaplan–Meier estimator, and the survival outcomes between the subphenotypes were compared with the log-rank test. A Cox proportional-hazards model was used to adjust the differences in survival between the subphenotypes for clinically important covariates.

Continuous variables were expressed as median and interquartile range (IQR) or mean and standard deviation (SD). Categorical variables were expressed as absolute frequencies and proportions. Differences between groups were assessed with the Chi-square test, Fisher’s exact test, Wilcoxon rank-sum test, and the t-test. Statistical significance was defined as p < 0.05. Longitudinal missing data for the clustering analysis was imputed using the Copy Mean method [26]. Cross-sectional missing data was imputed using multiple imputations with chained equations. All analyses were conducted with R (version 4.2.1).

## Results

During the study period from May 22, 2020, to February 10, 2022, a total of 802 patients were screened for eligibility, and 718 were included in the analysis. Of those, 504 were assigned to the development cohort and 214 to the validation cohort, based on a temporal split (Supplementary Fig. 1). In the development cohort, we used longitudinal data of the PaO_2_/FiO_2_, respiratory compliance_,_ and ventilatory ratio during the first prone session for the k-means clustering model. The optimal number of clusters was defined as two (termed subphenotypes A and B) based on quality metrics (Supplementary Fig. 3). The demographic and clinical characteristics of the development cohort are presented in Table 1. The median age of the patients was 60 years, with no significant difference between subphenotypes A and B. Female patients represented 42% of the total sample, with a higher proportion in subphenotype B (p < 0.001). The mean BMI was 30 kg/m^2^, with a higher value in subphenotype A (p = 0.05). Regarding comorbidities, 88% of patients had at least one. The most common comorbidities were arterial hypertension (59%) and diabetes (31%). A higher proportion of patients in subphenotype B had lung disease (p = 0.005). In terms of severity of disease, the overall median SAPS 3 score was 53, with a higher value in subphenotype B (p = 0.002). The overall mean SOFA score was 4, with a higher value in subphenotype B (p < 0.001).

**Table 1:** Demographic and clinical characteristics of the development cohort

	All	Subphenotype		
	N = 504^1^	A, N = 239^1^	B, N = 265^1^	p-value^2^
Demographics				
Age, years	60 (51, 69)	60 (51, 69)	61 (50, 70)	0.8
Sex, female	212 (42%)	65 (27%)	147 (55%)	< 0.001
BMI, kg/m^2^	30 (26, 35)	29 (26, 33)	30 (27, 36)	0.057
Comorbidities				
Any comorbidities	444 (88%)	211 (88%)	233 (88%)	> 0.9
Arterial hypertension	299 (59%)	135 (56%)	164 (62%)	0.2
Diabetes	156 (31%)	73 (31%)	83 (31%)	0.9
Heart disease	21 (4.2%)	8 (3.3%)	13 (4.9%)	0.4
Lung disease	22 (4.4%)	4 (1.7%)	18 (6.8%)	0.005
HIV/AIDS	12 (2.4%)	4 (1.7%)	8 (3.0%)	0.3
Kidney disease	6 (1.2%)	3 (1.3%)	3 (1.1%)	> 0.9
Cancer	5 (1.0%)	2 (0.8%)	3 (1.1%)	> 0.9
Severity				
SAPS 3	53 (46, 63)	50 (44, 61)	54 (47, 64)	0.002
SOFA	4.00 (2.00, 7.00)	3.00 (2.00, 6.00)	5.00 (3.00, 7.00)	< 0.001
Use of systemic steroids	491 (97%)	232(97%)	259(98%)	0.6
Non-invasive support before intubation	240 (48%)	139 (58%)	101 (38%)	0.001
Number of prone sessions	2.00 (1.00, 3.00)	2.00 (1.00, 3.00)	2.00 (1.00, 3.00)	0.15
Baseline ventilatory parameters				
Tidal volume/predicted body weight, ml/kg	6.31 (5.95, 6.92)	6.21 (5.94, 6.73)	6.34 (5.97, 7.07)	0.038
Respiratory rate, min	25.0 (22.0, 28.0)	24.0 (20.0, 26.0)	26.0 (24.0, 28.0)	< 0.001
Minute ventilation, L/min	10.00 (8.50, 11.00)	9.80 (8.60, 11.00)	10.00 (8.30, 11.00)	> 0.9
Arterial pH	7.31 (7.25, 7.37)	7.33 (7.27, 7.38)	7.29 (7.23, 7.36)	< 0.001
PCO_2_, mmHg	51 (45, 61)	48 (42, 57)	54 (48, 64)	< 0.001
Plateau pressure, cmH_2_O	24.0 (21.0, 26.0)	22.0 (20.0, 25.0)	25.0 (22.0, 28.0)	< 0.001
Positive end-expiratory pressure, cmH_2_O	10.00 (10.00, 12.00)	10.00 (10.00, 12.00)	10.00 (10.00, 12.00)	0.5
Driving pressure, cmH_2_O	13.0 (11.0, 16.0)	12.0 (10.0, 14.0)	14.0 (13.0, 17.0)	< 0.001
PaO_2_/FiO_2_, mmHg	121 (91, 147)	130 (103, 154)	111 (82, 142)	< 0.001
Compliance of respiratory system, cmH_2_O	29 (23, 35)	34 (30, 40)	24 (20, 29)	< 0.001
Ventilatory ratio	2.13 (1.76, 2.73)	1.96 (1.58, 2.34)	2.39 (1.95, 2.94)	< 0.001
Outcomes				
Hospital length-of-stay	17 (11, 28)	20 (12, 34)	15 (10, 24)	< 0.001
Ventilator-free days in 28-day survivors	3 (7)	4 (8)	2 (6)	< 0.001
28-day mortality	322 (64%)	125 (52%)	197 (74%)	< 0.001
60-day mortality	358 (71%)	146 (61%)	212 (80%)	< 0.001
90-day mortality	362 (72%)	149 (62%)	213 (80%)	< 0.001

^1^Median (IQR); n (%); Mean (SD)

^2^Wilcoxon rank sum test; Pearson’s Chi-squared test; Fisher’s exact test

**Fig. 1:**
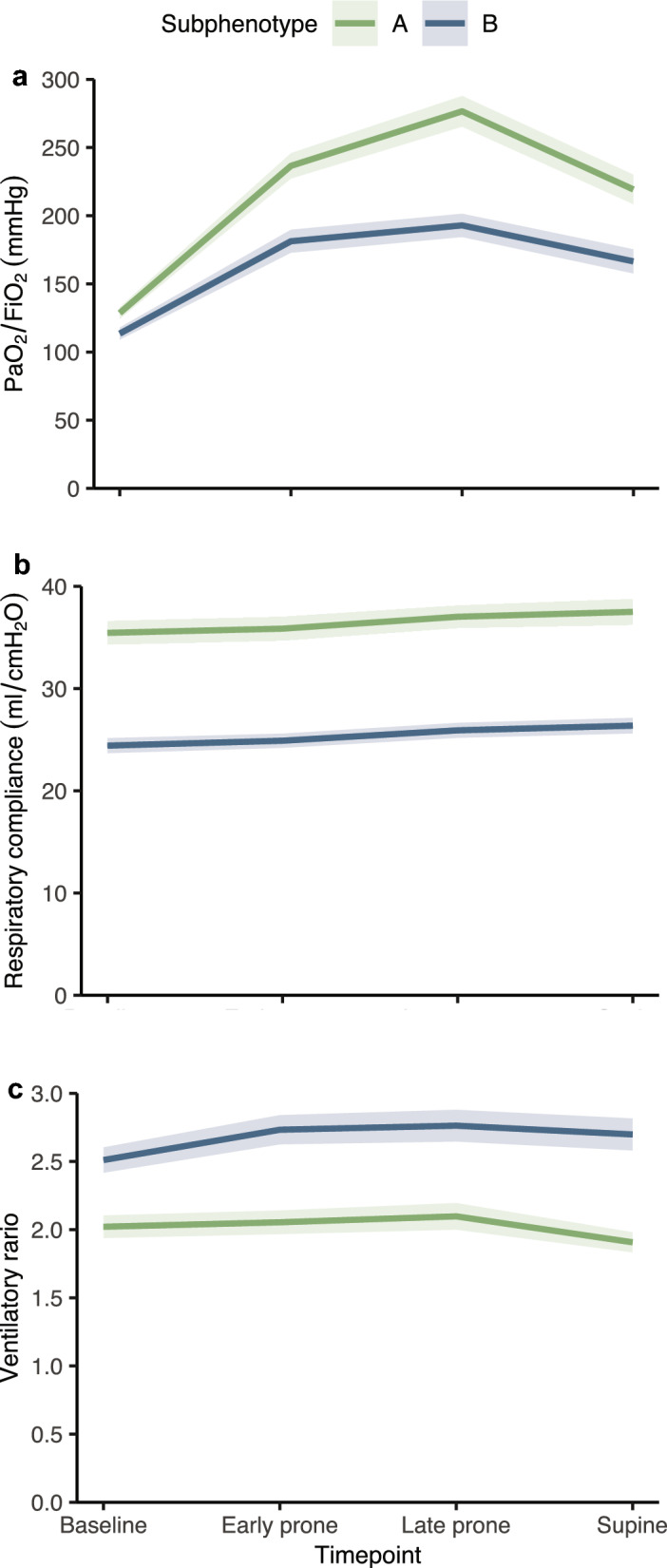
Trajectories of the clustering variables in subphenotypes A (green) and B (blue) in the development cohort. **a** PaO_2_/FiO_2_; **b** Respiratory compliance; **c** Ventilatory ratio. The lines represent the mean, and the bands represent the 95% confidence interval

**Fig. 2:**
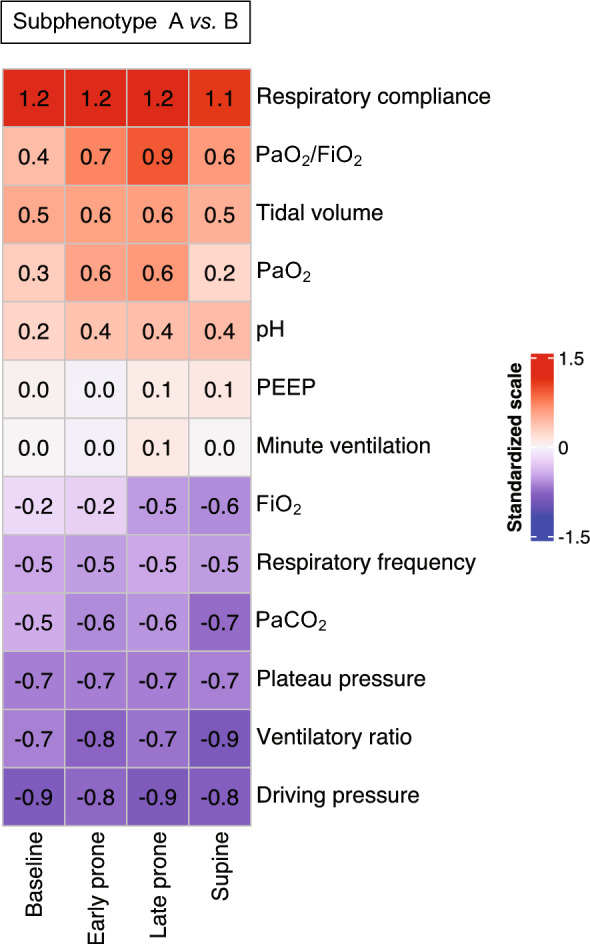
Differences in the respiratory variables between the subphenotypes. The heatmap represents the differences (subphenotype A minus B) of respiratory parameters (rows) at each of the measured time points during the prone session (columns). All variables were standardized so that the means correspond to 0 and the standard deviation correspond to 1

The subphenotypes had distinct trajectories of the classification variables. Notably, subphenotype A had higher respiratory compliance and PaO_2_/FiO_2_. In contrast, subphenotype B had a higher ventilatory ratio across all time points (Fig. 1). Sensitivity analyses using only the complete observations and only participants with a baseline PaO_2_/FiO_2_ < 150 from the development cohort yielded similar results (Supplementary Figs. 4 and 5). To further explore these contrasts, we calculated the standardized mean differences of all the available blood gas and ventilatory mechanics parameters between the subphenotypes (Fig. 2). The data from the parameters in the original scales is also available (Supplementary Table 1). Respiratory compliance showed the most remarkable difference between the groups throughout all time points, with a corresponding substantial difference in driving pressure. The greatest variation during the prone maneuver was in the PaO_2_/FiO_2_, partially sustained after returning to the supine position. The prone maneuver also influenced the ventilatory ratio and PaCO_2_ differences, albeit not as pronounced.

**Fig. 3:**
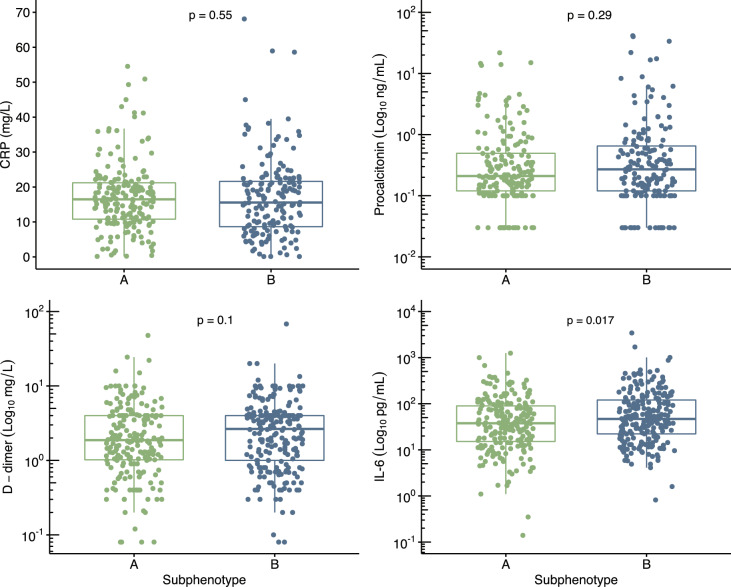
Comparison of thromboinflammation blood markers between the subphenotypes. The p-values were calculated using the Wilcoxon test

We also explored the association of thromboinflammation blood markers with the subphenotypes. The levels of IL-6 were significantly higher in subphenotype B (median: 47, IQR: 22–121) than in subphenotype A (median: 38, IQR: 15–90; p = 0.017), whereas CRP, procalcitonin, and D-dimer levels did not differ significantly between the groups (Fig. 3).

**Fig. 4:**
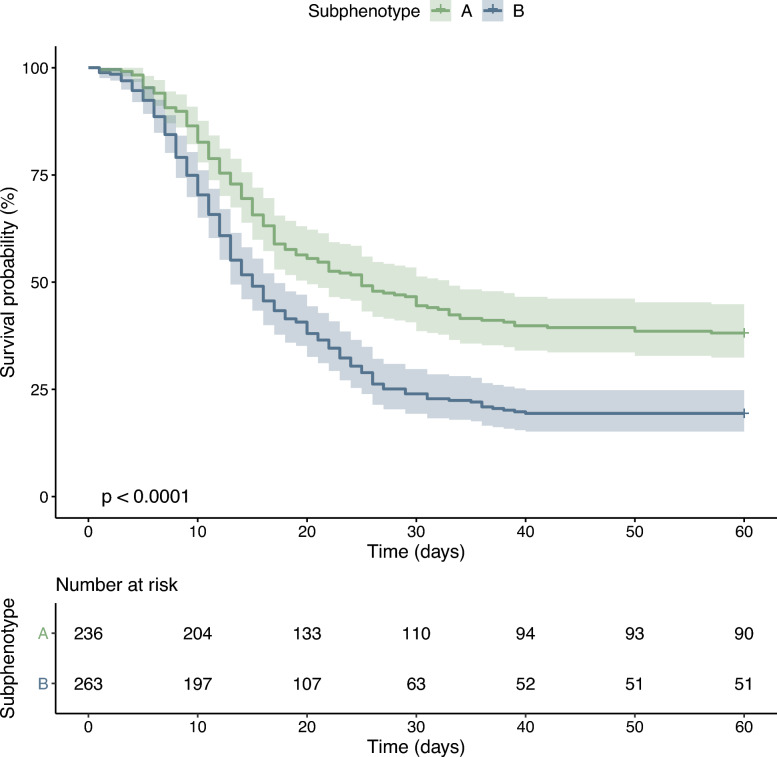
Kaplan–Meier plot of the probability of survival until day 60. The bands represent the 95% confidence intervals for point estimates of the survival curves. The p-values were calculated using the log-rank test

The subphenotypes were associated with different outcomes. Survival diverged early in the course between the groups. The difference was sustained over the observation period, reaching 20% in subphenotype B versus 39% in subphenotype A at 60 days (p < 0·0001) (Fig. 4). Additionally, the subphenotypes provided superior prognostic enrichment compared to classifications based on cross-sectional single parameters (Supplementary Fig. 6). After adjusting for age, sex, comorbidities, and obesity (BMI ≥ 30), subphenotype B was independently associated with an increased risk of 60-day mortality in the development cohort (HR 1.92, 95% CI 1.54–2.39, p < 0.001). Additionally, Subphenotype B was associated with a lower number of ventilator-free days on day 28 (mean: 4, SD: 8 in subphenotype A, and mean: 2, SD: 6 in subphenotype B; p < 0.001), and a lower hospital length of stay (median: 20, IQR: 12, 34 in subphenotype A, and median: 15, IQR: 10, 24 in subphenotype B; p < 0.001) (Table 1).

To test the reproducibility of the results, we trained a Random Forest model using the development cohort. The model was able to classify the subphenotypes with an accuracy of 95% (out-of-bag estimation of error of 4.76%) (Supplementary Fig. 7). The variables most important to the accurate distinction of the subphenotypes were the PaO_2_/FiO_2_ at late prone, respiratory compliance at baseline, ventilatory ratio at supine, and respiratory compliance at late prone. In contrast, the least important variable was the PaO_2_/FiO_2_ at baseline (Supplementary Fig. 8). We then used the model to classify the validation cohort. The demographic profile, trajectories of the classification variables, and survival analysis were similar to the development cohort (Supplementary Table 2 and Supplementary Fig. 9).

## Discussion

In this study, we present new subphenotypes of COVID-19 ARDS derived from a data-driven, multivariate analysis of the PaO_2_/FiO_2_, respiratory compliance, and ventilatory ratio in a prospective cohort. These key respiratory parameters were evaluated longitudinally before, during, and after the first prone session. We have shown the subphenotypes to be robust and consistent, as they could be accurately derived by a different classification model and validated on a separate cohort. A feature importance analysis demonstrated that all parameters and time points were relevant for the classification. Distinct clinical characteristics were observed for subphenotypes A and B. Subphenotype B is characterized by a lower oxygenation response, reduced compliance, and higher ventilatory ratio, reflecting more severe lung impairment and leading to a poorer prognosis. Furthermore, we explored the disparities and commonalities between these subphenotypes in a broader range of respiratory parameters and blood biomarkers. Lastly, we established an independent association between subphenotype B and a higher short-term mortality. The parameters incorporated in our model are readily available at the bedside and familiar to clinicians, consistent with standard respiratory monitoring practices. Additionally, the model’s low computational demands make it suitable for use on widely accessible devices, such as tablets, enabling real-time, bedside patient classification.

Longitudinal analyses can add a valuable dimension in which to differentiate ARDS patients. For example, a previous study systematically evaluated patients with COVID-19 ARDS and found no evidence for respiratory subphenotypes using single time points within the first four days of mechanical ventilation. Yet, a time-dependent analysis revealed a subphenotype with increasing ventilatory ratio associated with higher short-term mortality [21]. In our study, we evaluated a shorter but more dynamic timeframe. The prone maneuver acts as a challenge that reveals the differences among patients. This aspect is relevant when considering the importance of an early classification for prognostic and predictive enrichment purposes.

Improvement in oxygenation and reduction in mortality are the primary reasons for indicating the prone maneuver [12]. While a few studies suggest that the oxygenation response during a prone position may predict mortality in COVID-19 ARDS, the evidence is inconsistent across the ARDS literature [14, 15, 27, 28]. One methodological issue in many such studies is that O_2_ responders are defined by a somewhat arbitrary cutoff, usually an increase in the PaO_2_/FiO_2_ of at least 20 mmHg during the prone maneuver, which does not predict mortality in classical ARDS [28]. In COVID-19 ARDS, one study has shown a lower mortality in O2 responders, defined as having a PaO2/FiO2 variation at or above the median between the pre- and post-proning time points [14]. This finding suggests that classifications based on the underlying data distribution might be a better approach. Our study parsed the subphenotypes by a data-driven approach, considering all variables simultaneously. Although Subphenotype B had a markedly lower O_2_ response, the group would, on average, satisfy the O_2_ response criteria outlined above. Correspondingly, the baseline respiratory compliance was lower in subphenotype B, with a slight upward trajectory observed in both subphenotypes during the prone maneuver. Given the expected decrease in thoracic wall compliance in the prone position, these respiratory compliance trajectories suggest an increase in lung compliance, indicative of a net gain in alveolar recruitment [12]. Additionally, the higher and sustained ventilatory ratio in subphenotype B offers a more severe ventilation/perfusion mismatch that could not be reversed by the prone maneuver [7].

Many studies have sought to draw comparisons between COVID-19 and non-COVID-19-related ARDS, with mixed results [29]. Notably, our findings do not point to a phenotype with high compliance, low ventilation-to-perfusion ratio, and low recruitability, as previously suggested [30]. However, we cannot dismiss the possibility that such a phenotype may have been present in an earlier stage of disease. Pulmonary microthrombosis has been proposed as a key pathophysiological characteristic of COVID-19 [31]. Given this, it is interesting to ponder whether the consistently higher ventilatory ratio in subphenotype B might serve as a surrogate for COVID-19’s microvascular dysfunction [32]. This could suggest a potential benefit from targeted therapies such as anticoagulation in this group.

In contrast to the respiratory variables, the levels of C-reactive protein, procalcitonin, and D-dimer, commonly available blood biomarkers of thromboinflammation in clinical practice, did not differ between the subphenotypes in our study. However, the levels of IL-6, a pro-inflammatory biomarker associated with cytokine-release syndrome [33], were higher in subphenotype B. A previous study identified hypoinflammatory and hyperinflammatory subphenotypes in non-COVID-19 ARDS-based laboratory values and vital signs [34]. Interestingly, no clinically significant differences among these subphenotypes were identified in the respiratory variables. This suggests that a more specific panel of biomarkers may be necessary to capture the differences in the pulmonary pathophysiology across the subphenotypes observed in our study. It is also interesting to speculate that physiological and blood biomarker-based subphenotypes could have complementary roles in classifying ARDS patients.

This study has limitations. First, both the development and validation cohorts are from a single center. While a temporal split is not the optimal method for validation, it essentially functions as an external validation in the temporal dimension. Thus, it can be considered an intermediary method between internal and external validation [35]. Second, a PaO_2_/FiO_2_ of 200 mmHg or less was utilized as a selection criterion for the prone position, a more lenient cutoff than typically recommended in clinical practice [36]. To address this issue, we performed a sensitivity analysis using only patients with a PaO_2_/FiO_2_ of 150 mmHg or less, yielding similar results. Third, as this study only included patients with COVID-19 ARDS, the subphenotypes may not be generalizable for ARDS of other etiologies. Finally, determining the value of the identified subphenotypes for predictive enrichment was beyond the scope of this study.

## Conclusions

We have introduced two distinct subphenotypes of COVID-19 ARDS, each with unique physiological, clinical, and outcome characteristics. These subphenotypes have the advantage of being based on easily accessible variables, rendering them straightforward for clinicians to interpret and offering valuable insights into the heterogeneous nature of ARDS pathophysiology. Further research is needed to determine whether these subphenotypes could guide more targeted treatment approaches.

## Supplementary Information

Supplementary Material 1.

## Data Availability

The datasets used and analyzed during the current study are available from the corresponding author upon reasonable request.
