# Correction: Identification and validation of respiratory subphenotypes in patients with COVID-19 acute respiratory distress syndrome undergoing prone position

**DOI:** 10.1186/s13613-024-01417-9

**Published:** 2025-01-22

**Authors:** Monica R. da Cruz, Pedro Azambuja, Katia S. C. Torres, Fernanda Lima‑Setta, Andre M. Japiassu, Denise M. Medeiros

**Affiliations:** 1 Evandro Chagas National Institute of Infectious Diseases, Oswaldo Cruz Foundation (INI-Fiocruz), Rio de Janeiro, RJ Brazil; 2 Pedro Ernesto University Hospital (HUPE), Rio de Janeiro State University (UERJ), Rio de Janeiro, RJ Brazil; 3 National Institute of Women, Children and Adolescents Health Fernandes Figueira, Oswaldo Cruz Foundation (IFF-Fiocruz), Rio de Janeiro, RJ Brazil

**Correction: Annals of Intensive Care (2024) 14:178** 10.1186/s13613-024-01414-y

Following publication of the original article, the authors identified an error in Fig. 4. The current version with inverted labels would confuse readers as it contrasts with the results presented in the text.

Uncorrected Fig. 4.
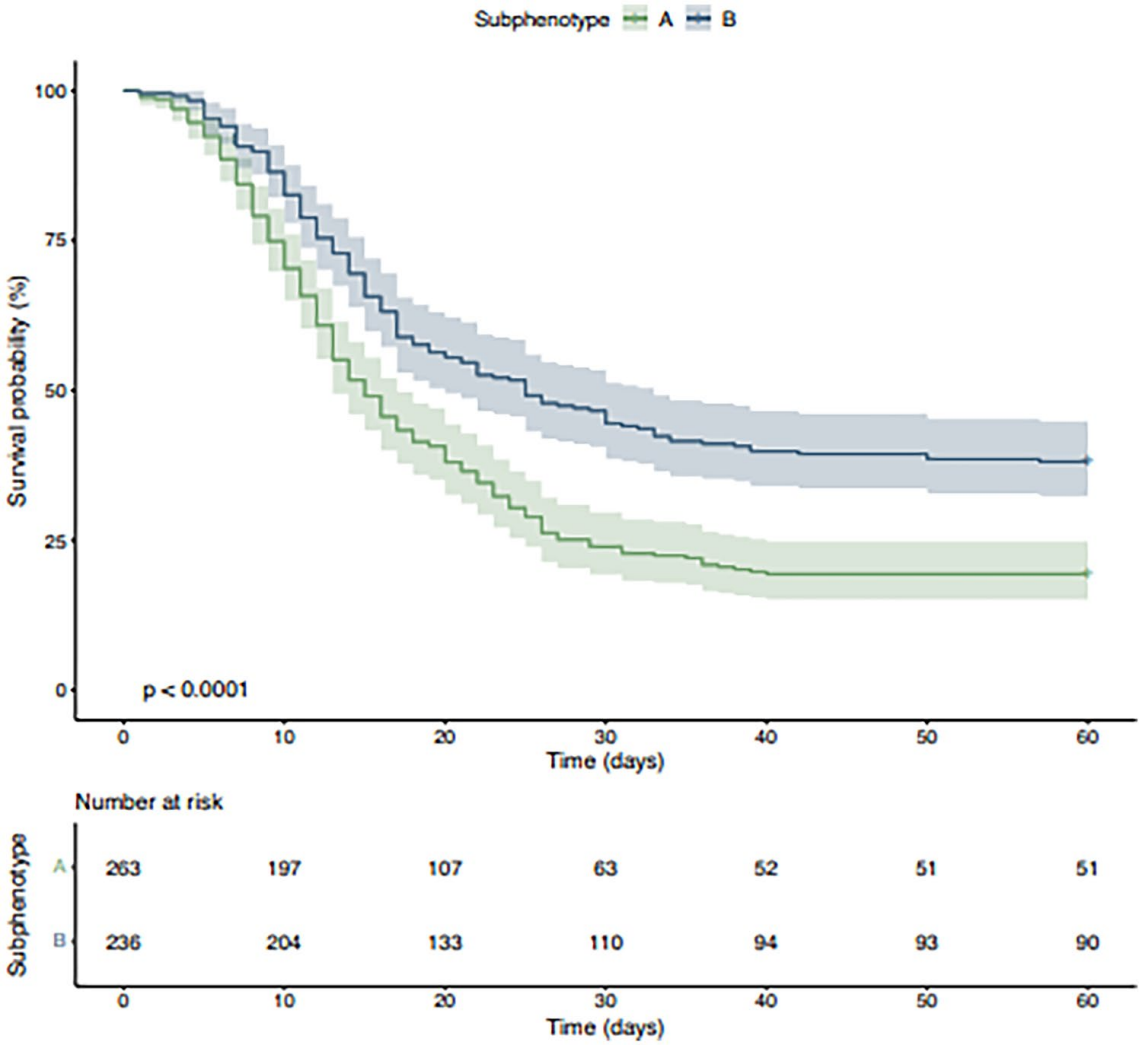


Corrected Fig. 4.

**Fig. 4:**
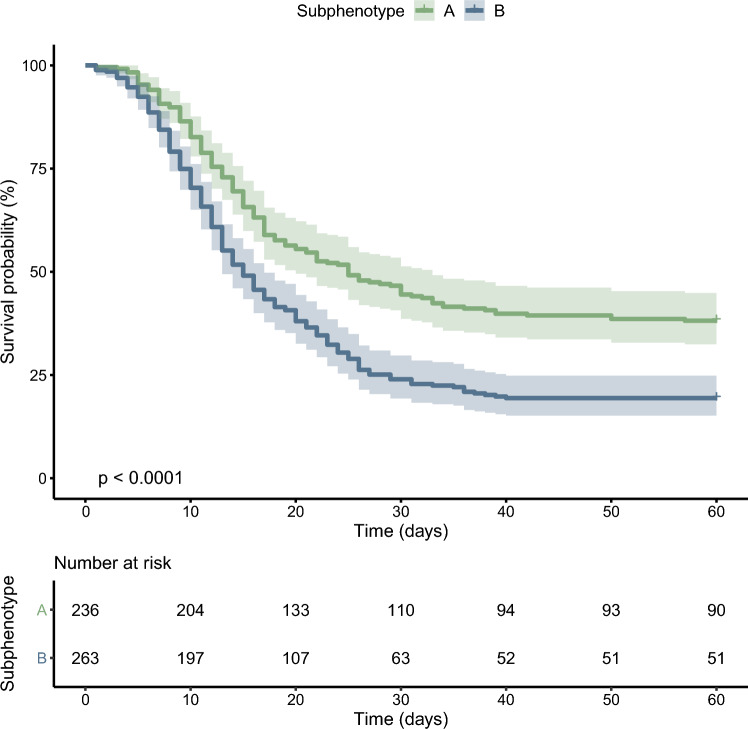
Kaplan–Meier plot of the probability of survival until day 60. The bands represent the 95% confidence intervals for point estimates of the survival curves. The p-values were calculated using the log-rank test

The original article has been corrected.
