# Supplementary material for: Identification and validation of respiratory subphenotypes in patients with COVID-19 acute respiratory distress syndrome undergoing prone position
Source: Ann Intensive Care. 2024 Nov 29;14:178. doi: 10.1186/s13613-024-01414-y (PMC11607308; doi:10.1186/s13613-024-01414-y)
Supplement: Supplementary file 1 — Supplementary Material 1. [file 13613_2024_1414_MOESM1_ESM.docx]

**Supplementary appendix**

**Identification and validation of respiratory subphenotypes in patients with COVID-19 acute respiratory distress syndrome undergoing prone position**

Mônica Rodrigues da Cruz, Pedro Azambuja, Kátia Silva Cavallaro, Fernanda Lima-Setta, André Miguel Japiassú, Denise Machado Medeiros

Table of Contents

[Supplementary methods 2](#_Toc167794561)

[Supplementary Table 1. Cluster stability analysis 3](#_Toc167794562)

[Supplementary Table 2. Cluster stability analysis 3](#_Toc167794563)

[Supplementary Fig. 1. Study flowchart 4](#_Toc167794564)

[Supplementary Fig. 2. Correlation matrix of respiratory parameters 5](#_Toc167794565)

[Supplementary Fig. 3. Selection of the number of clusters 6](#_Toc167794566)

[Supplementary Fig. 4. Sensitivity analysis 7](#_Toc167794567)

[Supplementary Fig. 5. Sensitivity analysis 8](#_Toc167794568)

[Supplementary Table 1. Characteristics of the ventilatory parameters stratified by subphenotype 9](#_Toc167794569)

[Supplementary Fig. 6. Short-term mortality in the development cohort utilizing different classification methods 10](#_Toc167794570)

[Supplementary Fig. 7. Out-of-bag error rate performance of the random forests model 11](#_Toc167794571)

[Supplementary Fig. 8. Feature importance analysis of the random forests model 12](#_Toc167794572)

[Supplementary Table 2. Demographic end clinical characteristics of the validation cohort 13](#_Toc167794573)

[Supplementary Fig. 9. Trajectories of the clustering variables 14](#_Toc167794574)

[Supplement references 15](#_Toc167794575)

# **Supplementary methods**

**Staff Training**

Considering the rapid need for multidisciplinary team readiness, all professionals were trained in person before starting to work in our hospital. The team was trained using training manikin to simulate an intubated patient. Additionally, an accompanying educational video was made available to staff, led by a respiratory therapist, showing all phases of completing the prone position (1). A checklist was performed to assure consistency in the prone position procedure (supplementary appendix), and a treatment guideline was established, adapted from the PROSEVA study (2), in consensus among the physician, nursing, and respiratory care team at our institution.

**Prone position protocol**

After eligibility, the team placed intubated subjects diagnosed with COVID-19 ARDS in the prone position. Patients were fully sedated (Richmond Agitation-Sedation Scale -5), and neuromuscular blocking was used to avoid ventilatory asynchronies before and during the protocol. Mean arterial blood pressure (MAP) was measured continuously, and fluids and vasopressors were provided to maintain mean arterial pressure above 60 mmHg. The prone position was maintained for at least 16 consecutive hours, except if adverse events occurred or cardiopulmonary resuscitation was needed. All patients were ventilated on both VCV or PCV modes and lung protective ventilation was applied as follows: Vt targeted at 6 ml/kg of predicted body weight, plateau pressure (Pplat) < 30 cmH_2_O, FiO_2_ was titrated to obtain a peripheral oxygen saturation (SpO_2_) between 92 and 95% and PEEP that keeps the minimal dP_aw_ was utilized for all subjects. Physiological variables and arterial blood gases were measured at predetermined times: 1-“Baseline,” within 1 hour before the start of the prone session; 2-“Early prone,” 1 hour after the beginning of the prone session; 3-“Late prone” on the last hour of the prone session; 4-“Supine,” 4 hours after the termination of the prone session. The criteria for stopping prone treatment were improvement in oxygenation when the patient was returned to supine (defined as a PaO_2_/FiO_2_ ≥180 mm Hg and a FiO_2_ of ≤0.6) or PaO_2_/FiO_2_ worsening during the prone position. Side changes from the swimmer's position were performed every 2 hours to avoid complications such as pressure ulcers and edema. Immediate interruption of the prone position included removing lines, tubes, and hemodynamic instability.

Supplementary Table 1. Cluster stability analysis for the longitudinal feature set

| **Metric** | **Cluster 1** | **Cluster 2** |
| --- | --- | --- |
| **Jaccard Coefficient** | 0.9341834 | 0.9325505 |
| **Dissolved** | 0 | 0 |
| **Recovered** | 1000 | 1000 |

Supplementary Table 2. Cluster stability analysis for the baseline feature set

| **Metric** | **Cluster 1** | **Cluster 2** | **Cluster 3** | **Cluster 4** |
| --- | --- | --- | --- | --- |
| **Jaccard Coefficient** | 0.8289879 | 0.8473291 | 0.7794216 | 0.8732217 |
| **Dissolved** | 62 | 20 | 38 | 2 |
| **Recovered** | 836 | 878 | 671 | 901 |

# Supplementary Fig. 1. Study flowchart

Supplementary Fig. 2. Correlation matrix of respiratory parameters. (A) All measured parameters. (B) The parameters utilized in the cluster analysis. The fill color and the numbers in each square represent the Pearson coefficient for each pair of parameters. Crs: Respiratory compliance.

Supplementary Fig. 3. Selection of the number of clusters by quality criterium (Calinski & Harabasz). The partitions are marked by their corresponding cluster number. The one marked by a black dot has the highest index and was selected.

Supplementary Fig. 4. Sensitivity analysis using only the complete cases of the development cohort (N = 392). (A) Distribution of the missing observations. The numbers between parenthesis represent the percentage of missing observations. (B) On the left is the selection of the number of clusters by the Calinski & Harabasz index. On the right are the trajectories of the clustering variables. P/F: PaO_2_/FiO_2_; Crs: Respiratory compliance; VR: ventilatory ratio.

Supplementary Fig. 5. Sensitivity analysis using only patients with a baseline PaO2/FiO2 < 150 of the development cohort (N = 387). (A) On the left is the selection of the number of clusters by the Calinski & Harabasz index. On the right are the trajectories of the clustering variables. P/F: PaO_2_/FiO_2_; Crs: Respiratory compliance; VR: ventilatory ratio. (B) Kaplan-Meier plot of the probability of survival until day 60. The p-value was calculated using the log-rank test.

# Supplementary Table 1. Characteristics of the ventilatory parameters stratified by subphenotype

| Characteristic | A, N = 239^1^ | B, N = 265^1^ | Difference^2^ | p-value^3^ |
| --- | --- | --- | --- | --- |
| **Ventilatory Ratio (Baseline)** | 1.96 (1.58, 2.34) | 2.39 (1.95, 2.94) | 0.43 | <0.001 |
| **Ventilatory Ratio (Early Prone)** | 1.95 (1.59, 2.44) | 2.60 (2.16, 3.26) | 0.65 | <0.001 |
| **Ventilatory Ratio (Late Prone)** | 1.97 (1.57, 2.49) | 2.57 (2.14, 3.28) | 0.60 | <0.001 |
| **Ventilatory Ratio (Supine)** | 1.89 (1.49, 2.32) | 2.56 (2.04, 3.20) | 0.67 | <0.001 |
| **Crs (Baseline), cmH2O** | 34 (30, 40) | 24 (20, 29) | -10.00 | <0.001 |
| **Crs (Early Prone), cmH2O** | 34 (29, 41) | 25 (21, 29) | -9.00 | <0.001 |
| **Crs (Late Prone), cmH2O** | 36 (31, 40) | 26 (22, 30) | -10.00 | <0.001 |
| **Crs (Supine), cmH2O** | 36 (31, 43) | 26 (22, 30) | -10.00 | <0.001 |
| **P/F Ratio (Baseline), mmHg** | 130 (103, 155) | 111 (82, 142) | -19.00 | <0.001 |
| **P/F Ratio (Early Prone), mmHg** | 237 (185, 286) | 172 (126, 227) | -65.00 | <0.001 |
| **P/F Ratio (Late Prone), mmHg** | 271 (216, 331) | 188 (138, 236) | -83.00 | <0.001 |
| **P/F Ratio (Supine), mmHg** | 204 (164, 263) | 159 (109, 209) | -45.00 | <0.001 |
| **Driving Pressure (Baseline), cmH2O** | 12.0 (10.0, 14.0) | 14.0 (13.0, 17.0) | 2.00 | <0.001 |
| **Driving Pressure (Early Prone), cmH2O** | 12.0 (10.0, 13.5) | 14.0 (12.0, 16.0) | 2.00 | <0.001 |
| **Driving Pressure (Late Prone), cmH2O** | 12.0 (10.0, 13.0) | 14.0 (12.0, 16.0) | 2.00 | <0.001 |
| **Driving Pressure (Supine), cmH2O** | 12.0 (9.0, 13.0) | 14.0 (12.0, 16.0) | 2.00 | <0.001 |
| **pH (Baseline)** | 7.33 (7.27, 7.38) | 7.29 (7.23, 7.36) | -0.04 | <0.001 |
| **pH (Early Prone)** | 7.32 (7.24, 7.38) | 7.27 (7.20, 7.35) | -0.05 | <0.001 |
| **pH (Late Prone)** | 7.34 (7.27, 7.41) | 7.30 (7.22, 7.37) | -0.04 | <0.001 |
| **pH (Supine)** | 7.37 (7.29, 7.44) | 7.32 (7.24, 7.39) | -0.05 | <0.001 |
| **Minute Ventilation (Baseline), L/min** | 9.80 (8.60, 11.00) | 10.00 (8.30, 11.00) | 0.20 | >0.9 |
| **Minute Ventilation (Early Prone), L/min** | 10.00 (9.00, 11.05) | 10.00 (8.70, 11.00) | 0.00 | 0.5 |
| **Minute Ventilation (Late Prone), L/min** | 10.00 (9.00, 11.95) | 10.00 (9.00, 11.60) | 0.00 | 0.3 |
| **Minute Ventilation (Supine), L/min** | 10.20 (9.00, 12.00) | 10.00 (9.00, 12.00) | -0.20 | 0.8 |
| **PCO2 (Baseline), mmHg** | 48 (42, 57) | 54 (48, 64) | 6.00 | <0.001 |
| **PCO2 (Early Prone), mmHg** | 49 (41, 59) | 58 (49, 70) | 9.00 | <0.001 |
| **PCO2 (Late Prone), mmHg** | 48 (40, 56) | 57 (48, 68) | 9.00 | <0.001 |
| **PCO2 (Supine), mmHg** | 44 (37, 53) | 54 (46, 64) | 10.00 | <0.001 |
| **PEEP (Baseline), cmH2O** | 10.00 (10.00, 12.00) | 10.00 (10.00, 12.00) | 0.00 | 0.5 |
| **PEEP (Early Prone), cmH2O** | 10.00 (8.00, 11.00) | 10.00 (8.00, 10.00) | 0.00 | >0.9 |
| **PEEP (Late Prone), cmH2O** | 10.00 (8.00, 12.00) | 10.00 (8.00, 12.00) | 0.00 | 0.043 |
| **PEEP (Supine), cmH2O** | 10.00 (10.00, 12.00) | 10.00 (8.00, 12.00) | 0.00 | 0.035 |
| **Plateau Pressure (Baseline), cmH2O** | 22.0 (20.0, 25.0) | 25.0 (22.0, 28.0) | 3.00 | <0.001 |
| **Ventilatory Ratio (Baseline)** | 22.0 (19.0, 24.0) | 24.0 (22.0, 27.0) | 2.00 | <0.001 |
| **Ventilatory Ratio (Early Prone)** | 22.0 (19.0, 24.0) | 24.0 (22.0, 27.0) | 2.00 | <0.001 |
| **Ventilatory Ratio (Late Prone)** | 21.0 (19.0, 24.0) | 25.0 (21.0, 27.0) | 4.00 | <0.001 |
| **Ventilatory Ratio (Supine)** | 24.0 (20.0, 26.0) | 26.0 (24.0, 28.0) | 2.00 | <0.001 |
| **Crs (Baseline), cmH2O** | 25.0 (22.0, 26.0) | 26.0 (24.0, 28.0) | 1.00 | <0.001 |
| **Crs (Early Prone), cmH2O** | 26.0 (24.0, 28.0) | 28.0 (25.0, 28.0) | 2.00 | <0.001 |
| **Crs (Late Prone), cmH2O** | 26.0 (24.0, 28.0) | 28.0 (25.0, 28.0) | 2.00 | <0.001 |
| **Crs (Supine), cmH2O** | 100 (70, 100) | 100 (80, 100) | 0.00 | 0.038 |
| **P/F Ratio (Baseline), mmHg** | 100 (70, 100) | 100 (80, 100) | 0.00 | 0.013 |
| **P/F Ratio (Early Prone), mmHg** | 60 (50, 70) | 70 (60, 90) | 10.00 | <0.001 |
| **P/F Ratio (Late Prone), mmHg** | 60 (50, 80) | 80 (60, 100) | 20.00 | <0.001 |
| **P/F Ratio (Supine), mmHg** | 103 (84, 126) | 92 (76, 115) | -11.00 | <0.001 |
| **Driving Pressure (Baseline), cmH2O** | 197 (142, 242) | 151 (109, 205) | -46.00 | <0.001 |
| **Driving Pressure (Early Prone), cmH2O** | 163 (131, 194) | 131 (99, 163) | -32.00 | <0.001 |
| **Driving Pressure (Late Prone), cmH2O** | 124 (94, 173) | 109 (82, 155) | -15.00 | 0.004 |
| **Driving Pressure (Supine), cmH2O** | 420 (376, 460) | 380 (340, 420) | -40.00 | <0.001 |
| **pH (Baseline)** | 420 (370, 450) | 380 (340, 410) | -40.00 | <0.001 |
| **pH (Early Prone)** | 420 (380, 456) | 380 (340, 420) | -40.00 | <0.001 |
| **pH (Late Prone)** | 420 (388, 457) | 390 (350, 422) | -30.00 | <0.001 |
| ^1^Median (IQR) |  |  |  |  |
| ^2^(B-A) |  |  |  |  |
| ^3^Wilcoxon rank sum test |  |  |  |  |

Supplementary Fig. 6. Short-term mortality in the development cohort utilizing different classification methods in the first prone session. (A) Subphenotypes A and B: Mortality of 125/239 (52%) in subphenotype A and 197/265 (75%) in subphenotype B (difference of 22 percentage points). (B) Median baseline (pre-prone) compliance: Mortality of 172/245 (70%) in the low compliance group and 150/259 (58%) in the high compliance group (difference of 12 percentage points). (C) Median baseline (pre-prone) ventilatory ratio: Mortality of 140/251 (56%) in the low ventilatory ratio group and 182/252 (70%) in the high ventilatory ratio group (difference of 16 percentage points). (D) Median P/F response, calculated as the percent change between the baseline (pre-prone) and 4 hours after re-supination: Mortality of 178/252 (71%) in the low P/F response group and 144/252 (57%) in the high P/F response group (difference of 14 percentage points). In B-D, Low: < median; High: ≥ median. The p-values were calculated using the log-rank test.

Supplementary Fig. 7. Out-of-bag error rate performance of the random forests model. The error rate is plotted against the number of decision trees included in the ensemble.

Supplementary Fig. 8. Feature importance analysis of the random forests model. Each variable is plotted according to two important measures: Mean Decrease Accuracy (left) and Mean Decrease Gini (right). Higher values suggest that a feature is more important for distinguishing between the classes.

# Supplementary Table 2. Demographic end clinical characteristics of the validation cohort

|  | **All** | **Subphenotypes** | | |
| --- | --- | --- | --- | --- |
|  | N = 214^1^ | **A**, N = 126^1^ | **B**, N = 88^1^ | **p-value^2^** |
| **Demographics** |  |  |  |  |
| Age, years | 58 (46, 70) | 54 (47, 67) | 66 (44, 74) | 0.057 |
| Sex, female | 81 (38%) | 28 (22%) | 53 (60%) | <0.001 |
| BMI, kg/m2 | 29 (26, 33) | 29 (25, 33) | 30 (27, 34) | 0.2 |
| **Comorbidities** |  |  |  |  |
| Any comorbidities | 206 (96%) | 123 (98%) | 83 (94%) | 0.3 |
| HIV/Aids | 8 (3.7%) | 4 (3.2%) | 4 (4.5%) | 0.7 |
| Arterial hypertension | 119 (56%) | 70 (56%) | 49 (56%) | >0.9 |
| Diabetes | 65 (30%) | 36 (29%) | 29 (33%) | 0.5 |
| Heart disease | 12 (5.6%) | 5 (4.0%) | 7 (8.0%) | 0.2 |
| Lung disease | 6 (2.8%) | 3 (2.4%) | 3 (3.4%) | 0.7 |
| Kidney disease | 5 (2.3%) | 1 (0.8%) | 4 (4.5%) | 0.2 |
| Cancer | 1 (0.5%) | 0 (0%) | 1 (1.1%) | 0.4 |
| **Severity** |  |  |  |  |
| SAPS 3 | 52 (45, 62) | 50 (44, 58) | 56 (48, 62) | 0.013 |
| SOFA | 4.00 (3.00, 6.00) | 4.00 (3.00, 5.75) | 5.00 (3.00, 7.00) | 0.15 |
| **Use of systemic steroids** | 213 (100%) | 126 (100%) | 87 (99%) | 0.4 |
| **Non-invasive support before intubation** | 120 (56%) | 79 (63%) | 41 (47%) | 0.019 |
| **Number of prone sessions** | 2.00 (1.00, 3.00) | 2.00 (1.00, 3.00) | 2.00 (1.00, 4.00) | 0.2 |
| **Baseline ventilatory parameters** |  |  |  |  |
| Tidal volume/predicted body weight, ml/kg | 6.17 (5.92, 6.89) | 6.06 (5.96, 6.63) | 6.39 (5.86, 6.95) | 0.2 |
| Respiratory rate, min | 25.0 (22.0, 28.0) | 24.0 (20.0, 26.0) | 26.0 (24.0, 28.0) | <0.001 |
| Minute ventilation, L/min | 9.70 (8.40, 10.80) | 10.00 (8.80, 11.17) | 9.40 (8.07, 10.50) | 0.015 |
| Arterial pH | 7.32 (7.27, 7.37) | 7.34 (7.29, 7.38) | 7.31 (7.25, 7.35) | 0.002 |
| PCO2, mmHg | 46 (40, 53) | 44 (37, 51) | 49 (43, 54) | <0.001 |
| Plateau pressure, cmH2O | 23.0 (21.0, 25.0) | 22.0 (20.0, 24.0) | 24.0 (22.0, 27.0) | <0.001 |
| Positive end-expiratory pressure, cmH2O | 10.00 (10.00, 10.00) | 10.00 (10.00, 10.00) | 10.00 (10.00, 10.00) | 0.5 |
| Driving pressure, cmH2O | 13.0 (11.0, 15.0) | 12.0 (10.0, 14.0) | 14.0 (13.0, 16.0) | <0.001 |
| PaO2/FiO2, mmHg | 131 (100, 160) | 140 (111, 162) | 121 (96, 151) | 0.007 |
| Compliance of respiratory system, cmH2O | 30 (24, 35) | 33 (30, 39) | 23 (20, 27) | <0.001 |
| Ventilatory ratio | 1.89 (1.57, 2.39) | 1.73 (1.42, 2.15) | 2.26 (1.83, 2.67) | <0.001 |
| **Outcomes** |  |  |  |  |
| Hospital length-of-stay | 17 (10, 29) | 20 (11, 32) | 15 (9, 26) | 0.044 |
| Ventilator-free days in 28-day survivors | 3 (7) | 4 (8) | 2 (5) | 0.033 |
| 28-day mortality | 130 (61%) | 69 (55%) | 61 (69%) | 0.032 |
| 60-day mortality | 149 (70%) | 81 (64%) | 68 (77%) | 0.042 |
| 90-day mortality | 151 (71%) | 81 (64%) | 70 (80%) | 0.016 |
| ^1^Median (IQR); n (%); Mean (SD) | | | | |
| ^2^Wilcoxon rank sum test; Pearson's Chi-squared test; Fisher's exact test | | | | |

Supplementary Fig. 9. Trajectories of the clustering variables in subphenotypes A (green) and B (blue) in the validation cohort. (a) PaO_2_/FiO_2_; (b) Respiratory compliance; (c) Ventilatory ratio. The lines represent the mean, and the bands represent the 95% confidence interval.

**Supplement references**:

1. (120) Posição Prona l Orientações Básicas - YouTube [Internet]. [citado 23 de janeiro de 2023]. Disponível em: https://www.youtube.com/watch?v=XV8j0E0dMNI

2. Guérin C, Reignier J, Richard JC, Beuret P, Gacouin A, Boulain T, et al. Prone Positioning in Severe Acute Respiratory Distress Syndrome. N Engl J Med. 6 de junho de 2013;368(23):2159–68.
